# Mannose-Binding Lectin 2 as a Potential Therapeutic Target for Hepatocellular Carcinoma: Multi-Omics Analysis and Experimental Validation

**DOI:** 10.3390/cancers15194900

**Published:** 2023-10-09

**Authors:** Hangyu Liao, Jun Yang, Yuyan Xu, Juncheng Xie, Ke Li, Kunling Chen, Jingyuan Pei, Qiong Luo, Mingxin Pan

**Affiliations:** 1 General Surgery Center, Department of Hepatobiliary Surgery II, Guangdong Provincial Research Center for Artificial Organ and Tissue Engineering, Guangzhou Clinical Research and Transformation Center for Artificial Liver, Institute of Regenerative Medicine, Zhujiang Hospital, Southern Medical University, Guangzhou 510000, China; lhy5@i.smu.edu.cn (H.L.); yj12310155@i.smu.edu.cn (J.Y.); 240973846@i.smu.edu.cn (Y.X.); 3200101013@i.smu.edu.cn (J.X.); leekecs@hotmail.com (K.L.); 1987454446@i.smu.edu.cn (K.C.); pjy0612@i.smu.edu.cn (J.P.); 2 Department of General Surgery, The First Hospital of Changsha, Changsha 410000, China; 3 Department of General Surgery, Affiliated Hengyang Hospital, Southern Medical University (Hengyang Central Hospital), Hengyang 421000, China

**Keywords:** hepatocellular carcinoma, mannose-binding lectin 2, tumor microenvironment, miR-34c-3p, immune regulation

## Abstract

**Simple Summary:**

This study focuses on understanding the role of mannose-binding lectin 2 (MBL2) in hepatocellular carcinoma (HCC) and its potential as a target for therapy. Our objective was to investigate the influence of MBL2 on the proliferation and metastasis of hepatocellular carcinoma using integrated multi-omics analysis and experimental validation, with a specific emphasis on MBL2-related microRNAs. The results showed that low levels of MBL2 were associated with poor prognosis in HCC patients. We also found that increasing MBL2 levels could directly inhibit HCC cell growth and spread. Furthermore, miR-34c-3p was found to be a regulator of MBL2 expression. These findings provide new insights into the development of HCC and suggest that increasing MBL2 levels could be a potential strategy for HCC treatment.

**Abstract:**

Mannose-binding lectin 2 (MBL2), a member of the multimeric lectin family, is crucial in immune regulation and tumor development. *MBL2* gene polymorphisms are associated with the risk and prognosis of various tumors, including hepatocellular carcinoma (HCC). Its functional role in HCC remains largely unclear. In this study, we aimed to identify whether *MBL2* is a key regulator and a potential therapeutic target for HCC. A bioinformatics analysis revealed close relationships among *MBL2* downregulation, the tumor-associated proliferation and metastasis pathway, and tumor immunosuppressive microenvironments. Lower expression of *MBL2* in HCC patients was linked to an unfavorable prognosis. A cell counting kit-8 assay, colony formation assay, transwell migration assay, and wound healing assay further confirmed that the overexpression of *MBL2* could directly inhibit the proliferation and metastasis of HCC. Moreover, *MBL2* expression was regulated by miR-34c-3p, as confirmed by the dual-luciferase reporter assay, thereby demonstrating tumor progression in HCC cells. Thus, our study offers the first comprehensive confirmation of the role of *MBL2* in the development of HCC through multi-omics analysis and experimental validation. Furthermore, miR-34c-3p was found to be an upstream mechanism of the downregulation of *MBL2* expression and could be a promising therapeutic target, expanding treatment options for patients with HCC.

## 1. Introduction

Primary liver cancer is the seventh most common cancer and the second leading cause of cancer-related deaths worldwide [1]. Hepatocellular carcinoma (HCC) is a major type of primary liver cancer, with most patients (70–85%) diagnosed at an advanced stage and an overall 5-year survival rate of <12% [2,3]. The 3-year recurrence rate among patients who have undergone surgical resection for HCC is approximately 50% [4]. Existing treatments for advanced liver cancer are limited. With an improved understanding of the mechanisms underlying HCC development and progression, better therapeutic strategies can be established.

Mannose-binding lectin (MBL) is a multimeric lectin that recognizes a wide array of pathogens independent of specific antibodies and initiates the lectin pathway for complement activation. Most mammals have two types of MBL (MBL-A and MBL-C, also known as MBL1 and MBL2, respectively), whereas human MBL is structurally similar to type C [5]. MBL2 is involved in innate immunity and immune regulation [6]. MBL2 activates a complement through the lectin pathway [7], recognizing and removing pathogens, such as microorganisms and viruses [8], apoptotic cells, and necrotic cells [9]. Defective *MBL* gene expression or decreased serum MBL levels can lead to defects in cytophagocytosis, which is associated with recurrent infection and an increased severity of infectious diseases, especially in immunocompromised individuals [10,11,12]. Although MBL2-related studies have mostly focused on infectious and autoimmune diseases, studies have increasingly shown that *MBL2* gene polymorphisms are implicated in the risk and prognosis of different types of tumors. As a specific protein synthesized and secreted by the liver, its role in HCC development is indispensable.

MBL2 serves as a crucial regulator of the innate immune response and represents a prime candidate for genetic association studies in cancer due to its genomic heterogeneity [13,14]. Polymorphisms in the *MBL2* gene ultimately result in decreased serum MBL2 levels, which could potentially diminish immune surveillance against epithelial malignancies [15,16,17]. Genetic variants in the 3′-UTR of *MBL2* could potentially influence the risk of breast cancer in African American women [18]. Furthermore, A case-control study found that *MBL2* gene polymorphisms were associated with HCC development in Chinese patients with hepatitis B virus (HBV)-related cirrhosis [19]. However, the functional role of MBL2 in HCC remains unknown.

In this study, we comprehensively clarified the function of MBL2 in HCC using multi-omics analyses based on various public databases and validated in vitro and in vivo experiments. We analyzed the correlation between MBL2 and tumor-infiltrating lymphocytes in the tumor microenvironment and the relationship between potential upstream microRNAs (miRNAs) and the dysregulation of MBL2. In addition, we identified the role of MBL2-related miR-34c-3p in HCC in vitro. These findings provide a basis for MBL2 as a target for replacement therapy.

## 2. Materials and Methods

### 2.1. Data Collection and Processing

Transcriptome sequencing data and clinical information from liver cancer samples were retrospectively obtained from the following publicly available datasets: The Cancer Genome Atlas (TCGA) database (https://portal.gdc.cancer.gov/ (accessed on 5 June 2022)), the China HBV-HCC cohort (https://www.biosino.org/node/project/detail/OEP000321 (accessed on 15 June 2022)), the International Cancer Genome Consortium (ICGC) database (https://dcc.icgc.org/ (accessed on 8 July 2022)), and the NCBI Gene Expression Omnibus (GEO) database (https://www.ncbi.nlm.nih.gov/geo/ (accessed on 13 September 2022)). Specifically, data related to miRNA expression in liver cancer and pan-cancer RNA sequencing (RNA-seq) data were collected from TCGA database. The following five liver HCC cohorts were collected for further analysis: TCGA-Liver Hepatocellular Carcinoma (TGCA-LIHC), ICGC-LIRI (Japan), HBV-HCC, GSE14520, and GSE116174. Normal human tissue datasets were collected from the Genotype-Tissue Expression (GTEx) Project [20] and the atlas of normal tissue expression (ANTE) database [21]. RNA sequencing data, including counts and fragments per kilobase million values, were consistently transformed into transcripts per kilobase million values.

### 2.2. Analyzing the Potential Downstream Mechanism of MBL2

We constructed a coexpression correlation analysis of MBL2 using the LinkedOmics [22] dataset and annotated it using the Gene Ontology-Biological Process (GO-BP) dataset, Kyoto Encyclopedia of Genes and Genomes (KEGG) pathways, and transcription factor targets generated using Gene Set Enrichment Analysis (GSEA). The Oncobox pathway databank (OncoboxPD, https://open.oncobox.com (accessed on 12 September 2023)) [23] was applied for further validation.

### 2.3. Cell Culture and Transfection

The human HCC cell lines BEL-7404, human hepatoma-derived 7 (Huh7), and immortalized human embryonic kidney cells (HEK293T) were purchased from the Cell Bank of the Chinese Academy of Sciences (Shanghai, China) and were cultured as per the established protocols [24]. All cells were cultured in Dulbecco’s modified Eagle’s medium (DMEM) supplemented with 10% fetal bovine serum (FBS) (Gibco, Carlsbad, CA, USA), 100 units/mL penicillin, and 100 mg/mL streptomycin sulfate at 37 °C with 5% CO_2_.

Human MBL2 cDNA was constructed and packaged into lentiviral vectors by Hanbio (Shanghai, China) using the component sequence pHBLV-U6-MCS-CMV-ZsGreen-PGK-puromycin. The experimental group overexpressing MBL2 and the negative control group were named LV-MBL2 and LV-Ctrl, respectively. The transfection of the MBL2 overexpression lentiviral vector and the negative control vector was performed in a biological safety cabin, following the manufacturer’s protocol. The miR-34c-3p mimic and the miR-34c-3p inhibitor were generated by GenePharma (Suzhou, China). The sequences of the miRNA mimics and inhibitors are listed in Supplementary Tables S1 and S2, respectively.

### 2.4. Western Blot Analysis

Protein expression levels were assessed using an immunoblot analysis of cell lysates (20–30 μg of protein) in a radioimmunoprecipitation assay buffer (Beyotime, Shanghai, China) containing 1× phosphate-buffered saline (PBS), 5 mM ethylenediamine tetraacetic acid, 1% Nonidet P-40, 0.1% sodium dodecyl sulfate, 1 mM sodium orthovanadate, and 0.5% sodium deoxycholate and protease inhibitors. Protein concentrations were determined using a bicinchoninic acid protein quantification kit (Beyotime). The polyvinylidene difluoride membranes were blocked with 5% bovine serum albumin and incubated at room temperature for 1 h. Primary antibodies against β-tubulin (1:1000, TA-10; ZsBio, Beijing, China) and MBL2 (1:1000, GTX132722; Genetex, Irvine, CA, USA) were used. Following overnight incubation at 4 °C, the polyvinylidene difluoride membranes were rinsed with PBS-Tween 20 and then exposed to horseradish peroxidase (HRP)-conjugated secondary antibodies (1:1 × 10^4^, ZB-2301 and ZB-2305; ZsBio) for 1 h at room temperature. Images were obtained using a Tanon-5200 chemiluminescent imaging system (Tanon Science & Technology, Shanghai, China). Three independent replicate experiments were performed. The quantitative analysis of the Western blots was performed using ImageJ software (version 2.14.0). The uncropped blots showing all the bands with molecular weight markers are depicted in the Supplementary Material.

### 2.5. RNA Isolation and Quantitative Real-Time Reverse Transcription Polymerase Chain Reaction Assay

Total RNA was isolated using a TRIzol reagent following the recommended protocol provided by the manufacturer. To quantify MBL2 expression, polyadenylated mRNA was purified from total RNA and subsequently used for reverse transcription. A quantitative polymerase chain reaction (qPCR) was performed using SYBR^®^ Green PCR master mix (4309155; Thermo Fisher Technology, Shanghai, China) on an ABI 7500HT system (Applied Biosystems, Foster City, CA, USA). Glyceraldehyde-3-phosphate dehydrogenase (GAPDH) was used as the endogenous control. All primers were synthesized by Tsingke (Tsingke Biotechnology Co., Ltd., Beijing, China). The RT-qPCR primer sequences were as follows: MBL2, 5′-GTATGGTGGCAGCGTCTTACTCAG-3′ (forward) and 5′-GAAGCCGTTGATGCCTGGAGAG-3′ (reverse); GAPDH, 5′-GGAGCGAGATCCCTCCAAAAT-3′ (forward) and 5′-GGCTGTTGTCATACTTCTCATGG-3′ (reverse). The expression level of each target gene was presented as a fold change relative to the control group and was calculated using the 2-ΔΔCT method for relative quantification.

### 2.6. Fluorescence and Immunofluorescence Assays

The cells were cultured on confocal dishes overnight, fixed with 4% paraformaldehyde for 30 min, and permeabilized with 0.1% Triton X-100 for 10 min. Subsequently, the slides were blocked with 10% bovine serum albumin at room temperature for 30 min, incubated with antibodies overnight at 4 °C, and then washed three times with PBS. The coverslips were then incubated with Alexa Fluor 594-conjugated secondary antibodies (Thermo Fisher Technology, Shanghai, China) for 1 h at room temperature and stained with diamidino-phenyl-indole (D1306; Invitrogen, Thermo Fisher Technology, Shanghai, China). The cells were visualized using an Olympus FluoView FV1000 confocal microscope (Olympus, Hamburg, Germany). The acquired images were subsequently analyzed using the Olympus FLUOVIEW version 4.2a viewer software.

### 2.7. Cell Counting Kit-8 Assay

Cells were seeded at a density of 1 × 10^3^ cells/well in 96-well plates containing 100 μL of DMEM supplemented with 10% FBS. The plates were then incubated at 37 °C under a 5% CO_2_ atmosphere for 6 h. Subsequently, 10 μL of cell counting kit (CCK)-8 solution was added to each well every 24 h. After a 2 h incubation with the CCK-8 solution, the absorbance of each sample was measured at 450 nm using a microplate reader (Varioskan LUX, Thermo Scientific, Waltham, MA, USA).

### 2.8. Colony Formation Assay

One thousand viable HCC cells were plated in six-well plates and incubated in DMEM for seven days to form colonies. The colonies were rinsed three times with PBS, fixed with 4% formaldehyde for 15 min, and subsequently stained with Giemsa staining solution (Solarbio, Beijing, China) for 30 min. After washing three times with distilled water, images of the colonies on each plate were recorded using a light microscope (CKX53; Olympus Corporation, Tokyo, Japan). Colonies were analyzed using ImageJ software, and the results of the three experiments were averaged.

### 2.9. Transwell^®^ Migration Assay

Cells (5 × 10^4^ cells/300 μL) suspended in a serum-free medium were placed in the upper chamber of each 8 mm pore Transwell^®^ chamber (Corning Star, Cambridge, MA, USA). The lower chamber was filled with DMEM containing 20% FBS as a chemoattractant. The cells were allowed to migrate through the porous membrane for 24 h at 37 °C. Subsequently, five randomly chosen microscopic fields (×200 magnification) were observed, and the cells were counted. A minimum of four chambers from three independent experiments were analyzed.

### 2.10. Wound Healing Assay

Cells were plated and grown in six-well plates. After reaching confluence, cells were cultured in serum-free DMEM. A straight line was drawn perpendicular to the bottom of the plate using a sterile pipette tip to create a wound. After washing away the unattached cells, the adherent cells were cultured in a complete medium at 37 °C under a 5% CO_2_ atmosphere [25]. The wound healing process was visualized using a microscope at 0 and 24 h after scratching.

### 2.11. Predicting Upstream Binding miRNA of MBL2

We used the TargetScan (www.targetscan.org (accessed on 18 April 2021)) [26] database and the DIANA tool (microT-CDS) (diana.e-ce.uth.gr/tools (accessed on 15 February 2022)) [27] to identify and predict upstream binding miRNAs for MBL2. The miRNAs identified in TargetScan with a context++ score percentile ≥ 95 were extracted for further analysis.

### 2.12. Dual-Luciferase Reporter Assay

To confirm the binding of miR-34c-3p to the 3′-untranslated regions (3′-UTR) of MBL2, wild-type (WT) and mutant (Mut) sequences of MBL2 were synthesized and cloned into the pmirGLO luciferase vector by Kidan Biosciences (Guangzhou, China). These reporters were named MBL2-WT and MBL2-Mut, respectively. The sequences of the constructed plasmids and the mutation sites are listed in Supplementary Table S3.

### 2.13. Animals

The nude BALB/C-nu mice used in this study were purchased from the Experimental Animal Center of Southern Medical University, certified by the Guangdong Provincial Bureau of Science. The nude BALB/C-nu mice were maintained in a barrier facility in racks that were filtered using a high-efficiency particulate air filter. The animals were fed an autoclaved laboratory rodent diet. A total of eight nude mice were randomly and equally assigned to two groups. The experimental group received subcutaneous injections of Huh7 cells overexpressing MBL2, and the control group received subcutaneous injections of Huh7 cells expressing the control vector. On day 16, all subcutaneous tumors were collected, and their volumes and masses were measured. All animal experiments were approved by the Laboratory Animal Ethics Committee of Zhujiang Hospital of Southern Medical University according to the guidelines for the ethical treatment of animals.

### 2.14. Immunohistochemistry Assay

Immunohistochemistry was performed to detect the expression of proteins in 2.5 μm sections of formalin-fixed, paraffin-embedded HCC tissues and adjacent non-cancerous tissues as described previously [15]. Antigen retrieval was performed using a citrate buffer. After blocking endogenous peroxidase activity and nonspecific antigens, the slides were incubated overnight with primary antibodies against MBL2 (1:1000, DF4152; Affinity, Shanghai, China) at 4 °C. Mayer’s hematoxylin was used for nuclear counterstaining. An HRP-conjugated secondary antibody and a diaminobenzidine staining kit (Zsbio, Beijing, China) were used. The sections were independently assessed by three pathologists. The assessment of protein expression levels involved the multiplication of the percent positivity score and the staining intensity score. The percent positivity of antigen staining ranged from 0 to 4, with 0 representing 0%, 1 indicating 1–25%, 2 denoting 26–50%, 3 signifying 51–75%, and 4 representing >75%. The staining intensity was assessed using the following scale: 0 (no staining), 1 (weak staining, faint yellow), 2 (moderate staining, light brown), and 3 (strong staining, brown). In cases where discrepancies occurred, the pathologists jointly re-evaluated the sections to reach a consensus.

### 2.15. Protein–Protein Interaction Network Construction

Due to their high reliability, we utilized the GeneMANIA [28] and STRING [29] websites to analyze protein–protein interaction networks related to MBL2, with a primary emphasis on physical interactions.

### 2.16. Relationship between MBL2 and Immune Cell Infiltration

Three approaches were employed to assess the association between MBL2 expression and immune cell infiltration in the TCGA-LIHC cohort. First, the TIMER 2.0 database (http://timer.cistrome.org/ (accessed on 12 April 2023)) was used to evaluate the association between MBL2 expression and immunosuppressive infiltration by cancer-associated fibroblasts (CAF) and T-regulatory cells (Tregs). Second, the ImmuCellAI platform (http://bioinfo.life.hust.edu.cn/ImmuCellAI/#!/ ImmuCellAI (accessed on 25 April 2023)) was used to compute the immune cell infiltration scores of TCGA-LIHC patients. Lastly, the tumor immune dysfunction and exclusion (TIDE) algorithm, proposed by Jiang et al. [30], was employed to model the tumor microenvironment, which included CAF infiltration, the interferon-gamma level, myeloid-derived suppressor cell (MDSC) infiltration, T-cell dysfunction, and the prevention of T-cell infiltration in tumors. Moreover, the correlation between MBL2 expression and immune-related immunosuppressive gene expression was analyzed.

### 2.17. Statistical Analysis

The data were analyzed using R version 4.1.2 and the R Bioconductor packages (R Foundation for Statistical Computing, Vienna, Austria). The normality and homogeneity of variance were assessed using the Shapiro–Wilk normality test and the Bartlett homogeneity test, respectively [31]. Nonparametric or parametric methods, including the Wilcoxon test, Kruskal–Wallis test, t-test, and one-way analysis of variance, were employed to compare differences between groups. The “survminer” R package determined each dataset’s best cutoff point for survival information. The Kaplan–Meier method was utilized to generate survival curves for the prognostic analysis, while log-rank tests were performed to assess differences between groups. A univariate Cox regression model was employed to calculate the hazard ratio (HR) for MBL2. Furthermore, the “meta HR” between different liver cancer cohorts was integrated using the “meta” package. The correlation coefficients were computed using both Spearman’s and Pearson’s correlation analyses. The “ggcor” package was utilized to visualize the correlations. In order to determine if MBL2 was an independent predictor, we included MBL2 and relevant clinical parameters in the analysis of a multivariate Cox regression mode. All statistical analyses were two-sided, and statistical significance was set at *p* < 0.05.

## 3. Results

### 3.1. MBL2 Gene Expression and Prognostic Significance

**Figure 1:**
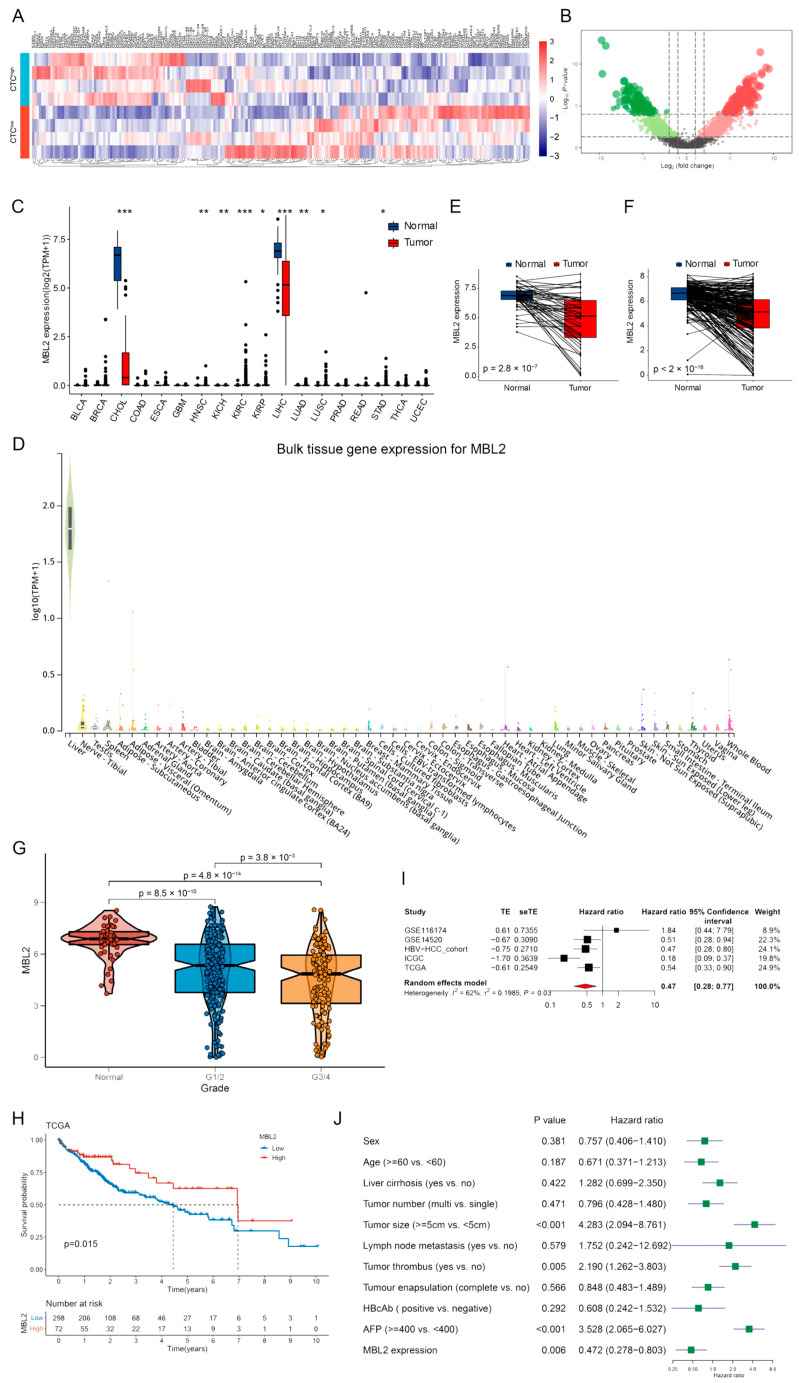
MBL2 gene expression and prognostic significance. (**A**) High-throughput sequencing was employed to compare transcriptional expression profiles between the two groups (CTC-high, CTC > 5; CTC-low, CTC = 0). (**B**) A heat map showing differential gene expression from high-throughput sequencing. (**C**) TCGA pan-cancer cohorts were used to explore MBL2 gene expression. (**D**) The GTEx database was utilized to confirm the basal expression of MBL2 in the liver tissue. (**E**,**F**) The mRNA expression of MBL2 in tumors and paired adjacent liver tissues from the TCGA and ICGC databases was analyzed. (**G**) The expression of MBL2 was assessed in normal liver tissue and HCC samples of different tumor grades. (**H**) Survival analysis of HCC patients based on the expression of MBL2 in the TCGA database. (**I**) The meta-analysis of the prognostic value of MBL2 expression in five HCC datasets is shown using a forest plot. (**J**) Univariate Cox regression analyses of MBL2 with other clinical parameters in the HBV-HCC cohort (* *p* < 0.05, ** *p* < 0.01, *** *p* < 0.001). MBL2, mannose-binding lectin 2; CTC, circulating tumor cell; TCGA, The Cancer Genome Atlas; GTEx, The Genotype-Tissue Expression Project; ICGC, International Cancer Genome Consortium; HBV-HCC, hepatitis B virus-related hepatocellular carcinoma.

Circulating tumor cells (CTCs) are closely associated with early HCC metastasis. To explore the mechanism of CTCs, six patients diagnosed with HCC were divided into two groups according to the preoperative peripheral blood CTC count (CTC-high, CTC > 5; CTC-low, CTC = 0). The transcriptional expression profiles of the two groups (Accession to Sequence Read Archive data: PRJNA912860) were compared using high-throughput sequencing (Figure 1A). In addition to the HCC-related cohort (GSE45436) in the GEO database, 82 CTC-related genes were screened. After a further analysis of immune-regulation-related genes, we successfully identified seven CTC-related immune regulation genes, and MBL2 was found to be significantly downregulated in high-CTC liver cancer tissues. Then, TCGA pan-cancer cohorts were used to explore MBL2 gene expression. MBL2 expression was significantly lower in liver cancer than in normal tissue and was hardly expressed in other tumors (Figure 1C). To confirm the inherent expression of MBL2 in liver tissue, we conducted an analysis of MBL2 expression in various normal tissues sourced from the Genotype-Tissue Expression (GTEx) and ANTE databases. We confirmed that MBL2 was specifically expressed in liver tissue (Figure 1D and Figure S1A). Compared with the paired adjacent liver tissue, the significant downregulation of MBL2 expression in HCC (Figure 1E,F and Figure S1B) indicates that the downregulated expression of MBL2 may play a critical role in promoting HCC.

We then analyzed the clinical characteristics and prognostic value of MBL2 expression in HCC. MBL2 expression was significantly upregulated in well-differentiated HCC samples compared to poorly differentiated samples (Figure 1G). Gao et al. [32] identified three subgroups of patients with HBV-related HCC based on proteomic data (the metabolism subgroup (S-Mb), dysregulated microenvironment subgroup (S-Me), and proliferation subgroup (S-Pf)), of which S-Pf harbored the strongest cell renewal pathways, such as cell cycle, transcription, splicing, and ubiquitin proteolysis, and had the worst prognosis. Our findings revealed that MBL2 expression was significantly lower in the S-Pf subgroup compared to the two other subgroups (Figure S1C).

To investigate the prognostic value of MBL2, three mRNA sequencing databases (TCGA-HCC, ICGC-HCC, and HBV-HCC cohorts) were constructed for a Kaplan–Meier analysis. Patients with low MBL2 expression showed significantly reduced overall survival compared with those with high expression (Figure 1H and Figure S1D,E). To validate the prognostic value of MBL2, we combined the transcriptomics sequencing cohorts with two other microarray expression profiling datasets (GSE116174 and GSE14520) from the GEO database. We performed a meta-analysis using the “meta” R package. The results revealed that the pooled HR was 0.47 (95% confidence interval: 0.28–0.77) with measurable heterogeneity (I^2^ = 62%, *p* = 0.03) and was adjusted by the random-effects model among the five datasets (Figure 1I). Furthermore, univariate and multivariate Cox regression analyses were performed to assess the relationships between MBL2 expression and other clinical features in the HBV-HCC cohort. The results revealed that low MBL2 expression served as an independent prognostic risk factor (Figure 1J and Figure S1F). We concluded that MBL2 is a prognostic risk factor that significantly influences the overall survival of patients with HCC.

### 3.2. Analysis of the Potential Downstream Mechanism of MBL2

**Figure 2:**
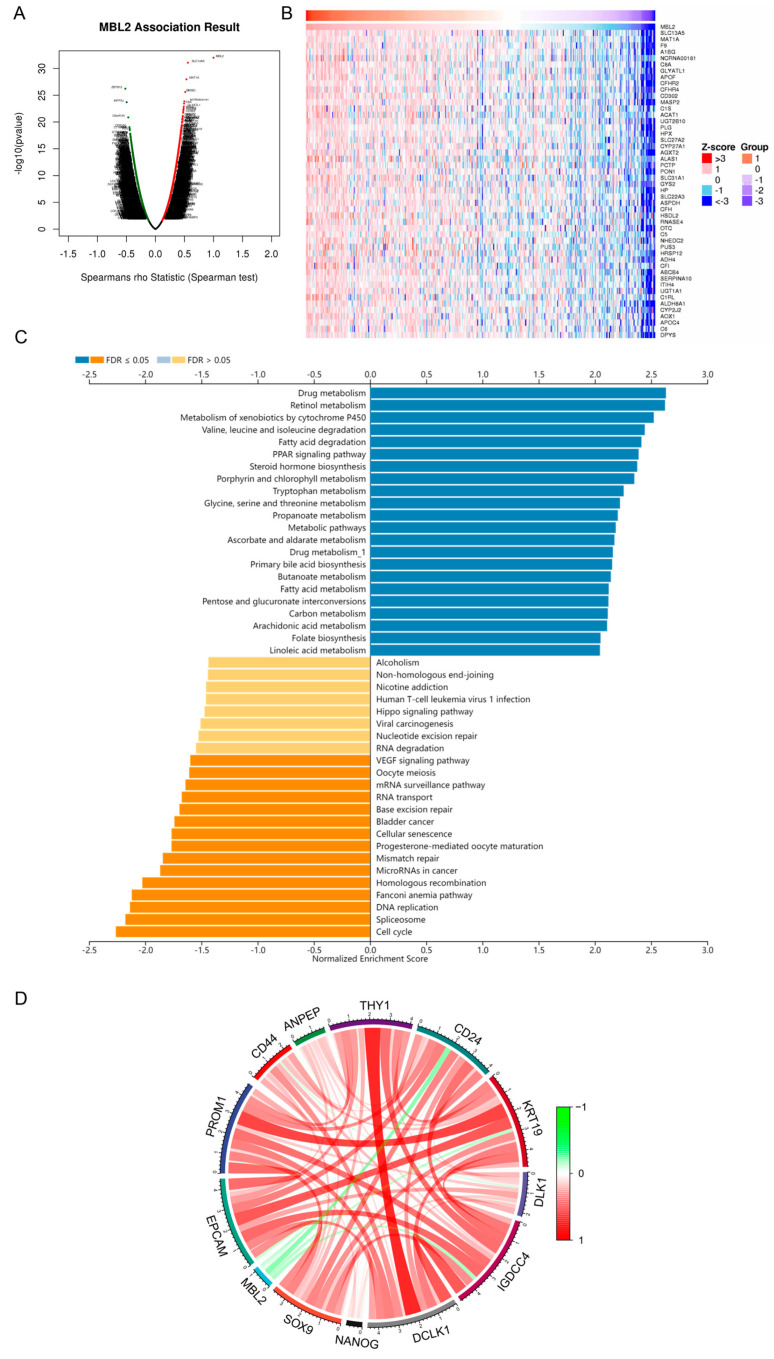
Analysis of the potential downstream mechanism of MBL2. (**A**) Genes highly coexpressed with MBL2 were identified through Spearman’s correlation tests in the TCGA-LIHC cohort. (**B**) Heat maps illustrating the top 50 genes that exhibit positive correlation with MBL2. (**C**) Significantly enriched gene ontology biological process annotations in HCC. (**D**) Spearman’s correlation analysis was conducted to examine the relationship between MBL2 expression and the expression of cancer stemness markers in the TCGA-LIHC dataset. The red connections represent positive correlations, and the green connections represent negative correlations. The color depth of a line represents the correlation coefficient. MBL2, mannose-binding lectin 2; TCGA-LIHC, The Cancer Genome Atlas-Liver Hepatocellular Carcinoma; HCC, hepatocellular carcinoma.

To explore the latent downstream mechanism by which MBL2 influences HCC progression, we constructed a coexpression correlation analysis of MBL2 using the LinkedOmics dataset (Supplementary Table S4). We found 7285 genes positively correlated with MBL2 expression, and 12,637 genes were negatively correlated (Figure 2A). The 50 genes with the strongest positive and negative correlations are shown in Figure 2B and Figure S2A, respectively. The coexpression gene list of MBL2 was then analyzed using the GO-BP terms, KEGG pathway, and transcription factor targets generated using GSEA. The results showed that HCC with low MBL2 expression was mainly involved in the cell cycle, DNA replication, miRNAs in cancer, and the vascular endothelial growth factor signaling pathway of the KEGG pathway (Figure 2C) and was enriched in GO-BP terms related to DNA replication, cell cycle checkpoints, chromatin remodeling, and the cell cycle G1/S phase transition (Figure S2B).

In contrast, HCC with high MBL2 expression was mainly involved in metabolism-related pathways (Figure 2C and Figure S2B), including drug, retinol, fatty acid metabolism, and steroid metabolic processes. A transcription factor target analysis indicated that HCC with low MBL2 expression was associated with the E2F family, whereas almost no transcription factor targets were enriched in those with high MBL2 expression (Figure S2C). The pathway activation level (PAL) calculated using the OncoboxPD revealed similar results (Figure S2D). A correlation analysis showed that MBL2 was significantly negatively corr elated with cancer stem cell-related markers (Figure 2D), such as NANOG and SOX9. Increased expression of BAX and a reduced BCL-2/BAX ratio may indicate a pro-apoptotic stimulus [33]. We conducted an analysis of the differential expression of apoptosis-related biomarkers between distinct MBL2 expression cohorts in the TCGA-LIHC dataset. However, among the apoptotic biomarkers, only the pro-apoptotic protein BAX and the death receptor Fas (FAS) showed significant differential expression between the various MBL2 expression cohorts (Figure S2E).

### 3.3. MBL2 Inhibits HCC Proliferation, Migration, and Invasion In Vitro

**Figure 3:**
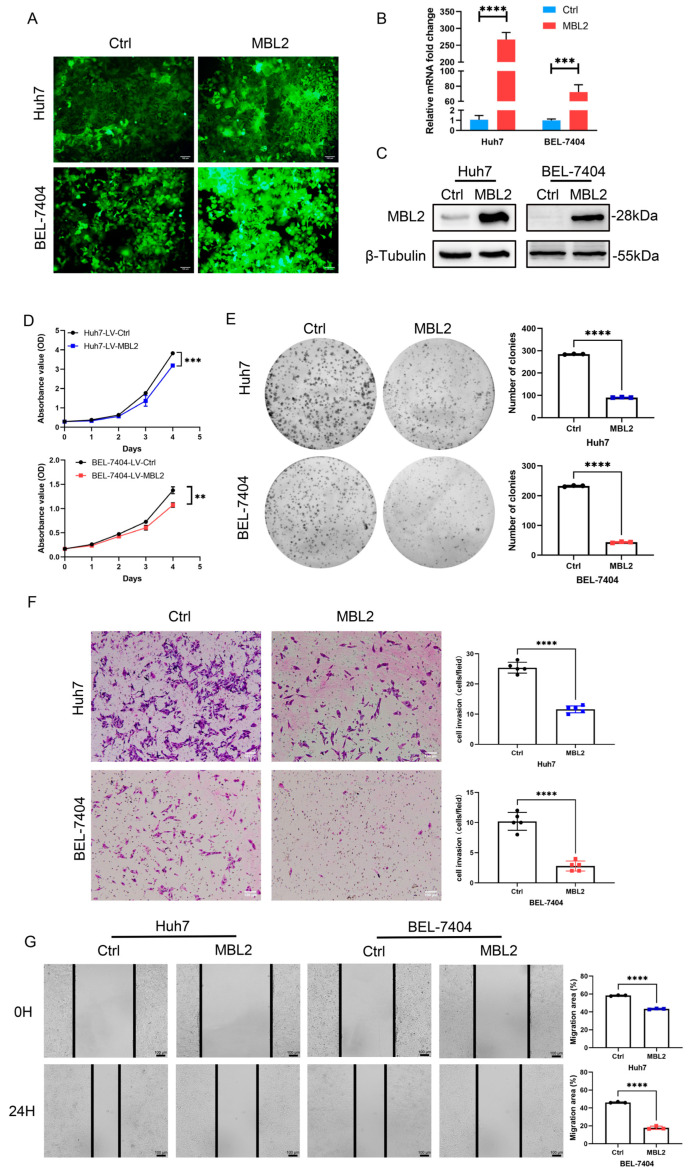
MBL2 inhibits HCC proliferation, migration, and invasion in vitro. (**A**) Fluorescence was used to show the transfection efficiency of lentiviruses carrying the MBL2 gene and the green fluorescent protein tag. (**B**,**C**) qPCR and Western blot assays were conducted to validate the successful establishment of MBL2 overexpression in Huh7 and BEL-7404 cell lines. (**D**,**E**) CCK-8 (**D**) and plate colony assays (**E**) were used to evaluate the effects of MBL2 on suppressing HCC proliferation. (**F**,**G**) Transwell (**F**) and wound healing assays (**G**) were used to assess the inhibitory effects of MBL2 on HCC migration and invasion (** *p* < 0.01, *** *p* < 0.001, **** *p* < 0.0001). MBL2, mannose-binding lectin 2; HCC, hepatocellular carcinoma; CCK-8, cell counting kit-8.

To verify the transfection efficiency of the MBL2 lentivirus, it was loaded with the MBL2 gene with a green fluorescent protein tag. A fluorescence analysis showed that Huh7 and BEL-7404 were successfully transfected with the lentivirus (Figure 3A). To verify the stable construction of the MBL2 gene, we used Western blot and qPCR assays to detect MBL2 expression at the transcriptional and translational levels, respectively (Figure 3B,C)**.** The calculated intensity ratios for the Western blot bands are presented in Figure S3A. The results showed that strains stably overexpressing MBL2 were successfully constructed in the Huh7 and BEL-7404 cell lines. CCK-8 and plate cloning experiments were performed to verify the regulatory effect of MBL2 on the proliferation of liver cancer cells (Figure 3D,E). The growth of the LV-MBL2 group was suppressed by the growth of the LV-Ctrl group. To verify the effect of MBL2 on the invasive ability of liver cancer cells, we used Transwell^®^ and wound healing assays (Figure 3F,G). The metastatic ability of the LV-MBL2 group was significantly lower than that of the LV-Ctrl group. In conclusion, MBL2 inhibited the proliferation, metastasis, and invasion of HCC cells in vitro.

### 3.4. miR-34c-3p Is Overexpressed in HCC and Promotes HCC Progression by Targeting MBL2

**Figure 4:**
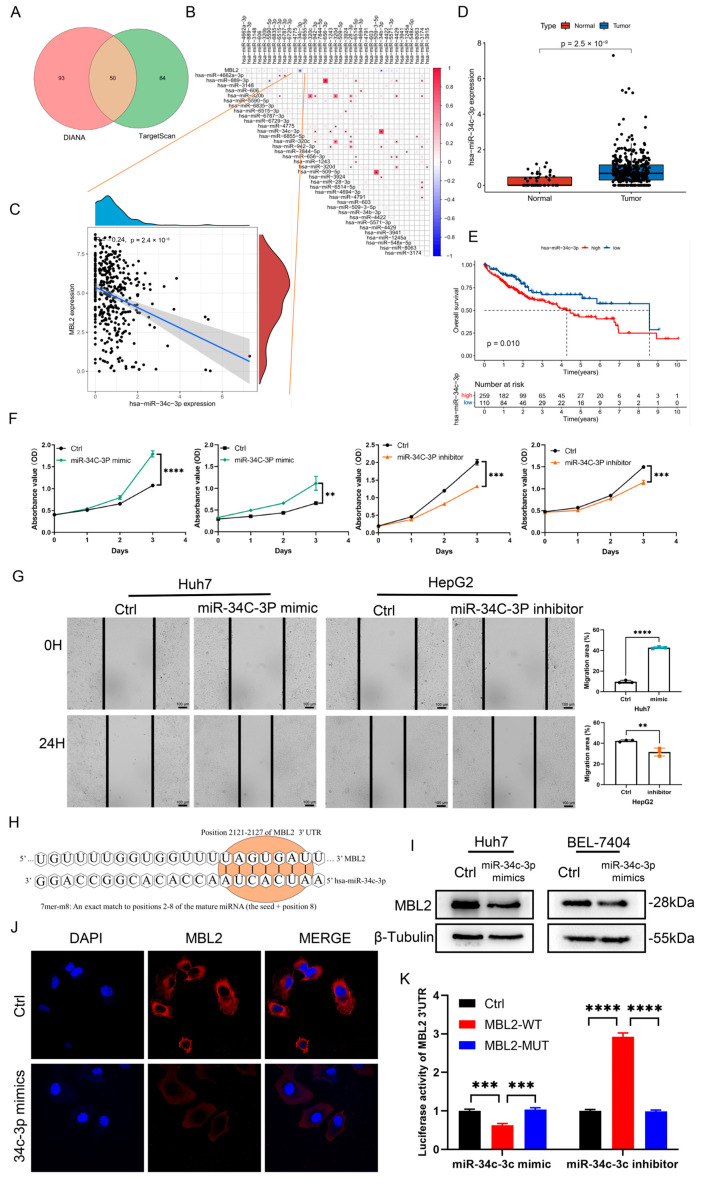
miR-34c-3p is overexpressed in HCC and promotes biological functions in vitro. (**A**) Venn diagram displaying the possible overlap of miRNAs of MBL2 between the TargetScan dataset and DIANA tool. (**B**) Analysis of correlation between MBL2 and upstream miRNA expression using Spearman’s analysis based on the TCGA-LIHC dataset. (**C**) Analysis of correlation between MBL2 expression and miR-34c-3p. (**D**) Expression of miR-34c-3p in HCC samples and liver tissue from the TCGA-LIHC cohort. (**E**) Kaplan–Meier survival analysis of HCC patients with high or low expression of miR-34c-3p based on the TCGA-LIHC cohort. (**F**) Gain- and loss-of-function analyses were used to study the effects of MBL2 on HCC cell proliferation as detected with a CCK-8 assay. (**G**) Gain- and loss-of-function analyses were used to study the effects of MBL2 on HCC cell migration as visualized using a wound healing assay. (**H**) Western blot analysis was performed to detect the expression of MBL2 in HEK293T. (**I**) Schematic representation of the potential binding sites for miR-34c-3p within 3′-UTRs of MBL2. (**J**) Immunofluorescence assay was used to examine the regulation of miR-34c-3p by MBL2 in Huh7 cells. (**K**) Dual-luciferase reporter confirmed the interaction between miR-34c-3p and MBL2 (** *p* < 0.01, *** *p* < 0.001, **** *p* < 0.0001). HCC, hepatocellular carcinoma; MBL2, mannose-binding lectin 2; HEK293T, immortalized human embryonic kidney cells; 3′-UTRs, three untranslated regions; Huh7, human hepatoma-derived 7; TCGA-LIHC, The Cancer Genome Atlas-Liver Hepatocellular Carcinoma; CCK-8, cell counting kit-8; Ctrl, control group.

These results preliminarily confirmed that MBL2 plays a crucial role in HCC progression. However, the cause of the aberrant downregulation of MBL2 in HCC remains unknown. miRNAs can regulate the expression of target genes by binding to their target gene mRNA and promoting its degradation. To explore the potential mechanism underlying the dysregulation of MBL2, we investigated potential upstream miRNAs that regulate MBL2 expression using the TargetScan and DIANA websites. Consequently, we identified 50 potential common upstream miRNAs (Figure 4A). Subsequently, using miRNA expression data from TCGA-LHC, we performed a Spearman’s correlation analysis of MBL2 and common miRNAs (Figure 4B), which revealed a noteworthy negative correlation between MBL2 and miR-34c-3p (Figure 4C). Moreover, the expression of miR-34c-3p was considerably upregulated in the liver compared with that in HCC (Figure 4D). Furthermore, the Kaplan–Meier survival analysis demonstrated that patients with high expression of miR-34c-3p exhibited significantly shorter overall survival than those with low expression (Figure 4E). To analyze the effect of miR-34c-3p on HCC progression, a sequence of miR-34c-3p mimics and miR-34c-3p inhibitors was constructed. CCK-8 assays were performed to confirm the regulatory effect of miR-34c-3p on liver cancer cell proliferation (Figure 4F). The results showed that the growth of the miR-34c-3p mimic group was markedly increased compared with that of the control group in the Huh7 and BEL-7404 cell lines. The growth of the miR-34c-3p inhibitor group was consistently suppressed by the negative control group in the HepG2 and Hep3B cell lines. To analyze the effect of miR-34c-3p on the migration ability of liver cancer cells, wound healing assays were performed on the HepG2 and Huh7 cell lines (Figure 4G). The results showed that the metastatic ability of the miR-34c-3p inhibitor group was significantly lower than that of the negative control group and vice versa. In conclusion, miR-34c-3p promotes the biological functions of HCC cells in vitro.

To further verify the miR-34c-3p and MBL2 interaction, we used the TargetScan database to predict the targets of miR-34c-3p acting on MBL2. A schematic representation (Figure 4H) illustrates the potential binding sites for miR-34c-3p within the 3′-UTRs of MBL2. To analyze the relationship between high expression of miR-34c-3p and the dysregulation of MBL2, miR-34c-3p mimics and inhibitors were transfected into the Huh7 and BEL-7404 cell lines. Western blotting was performed to detect MBL2 expression. Compared with the control group, the translational protein level of MBL2 in the miR-34c-3p mimic group was markedly decreased (Figure 4I and Figure S3B). A dual-luciferase reporter vector containing the WT or Mut 3′-UTR of MBL2 was co-transfected with an miR-34c-3p mimic or inhibitor in the HEK293T cell line. The luciferase activity of the WT 3′-UTR of MBL2 was decreased by the miR-34c-3p mimic but was increased by the miR-34c-3p inhibitor (Figure 4K). However, the luciferase activity of the Mut 3′-UTR of MBL2 was not affected by the miR-34c-3p mimic or inhibitor in HEK293T cells. To examine the regulation of MBL2 by miR-34c-3p in HCC cells, an immunofluorescence assay was used to explore the expression of MBL2 in Huh7 cells transfected with an miR-34c-3p mimic, showing that the expression of MBL2 was decreased by the miR-34c-3p mimic (Figure 4J).

### 3.5. Overexpression of MBL2 Inhibits HCC Growth In Vivo

**Figure 5:**
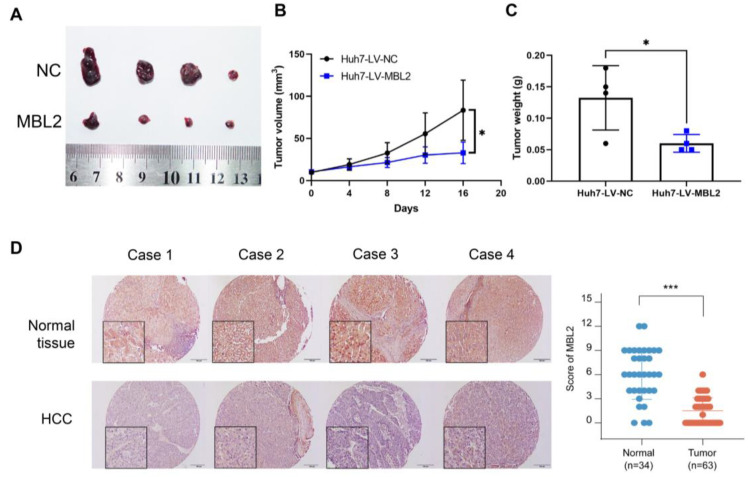
Overexpression of MBL2 inhibits HCC growth in vivo and its relationship with clinical cancer stage. (**A**) Nude mice were subcutaneously injected with Huh7 cells stably overexpressing MBL2. (**B**,**C**) The volumes and weights of the subcutaneous tumors showed that the overexpression of MBL2 inhibits subcutaneous tumor formation in nude mice. (**D**) The level of MBL2 was lower in liver cancer tissue compared to normal liver tissues (* *p* < 0.05, *** *p* < 0.001). MBL2, mannose-binding lectin 2; HCC, hepatocellular carcinoma; Huh7, human hepatoma-derived 7.

To investigate the effect of MBL2 on HCC cell growth in vivo, we constructed a subcutaneous tumor model in nude mice (Figure 5A). Tumor growth curves (Figure 5B) and weights (Figure 5C) showed that tumor growth was inhibited in the MBL2 group relative to that in the control group. Subsequently, we evaluated the expression level of MBL2 in liver cancer and adjacent normal tissues of liver cancer patients using immunohistochemistry (Figure 5D). The expression of MBL2 was markedly lower in liver cancer tissues compared to non-cancerous tissues.

### 3.6. MBL2 Expression Is Associated with Immune Infiltration of HCC

**Figure 6:**
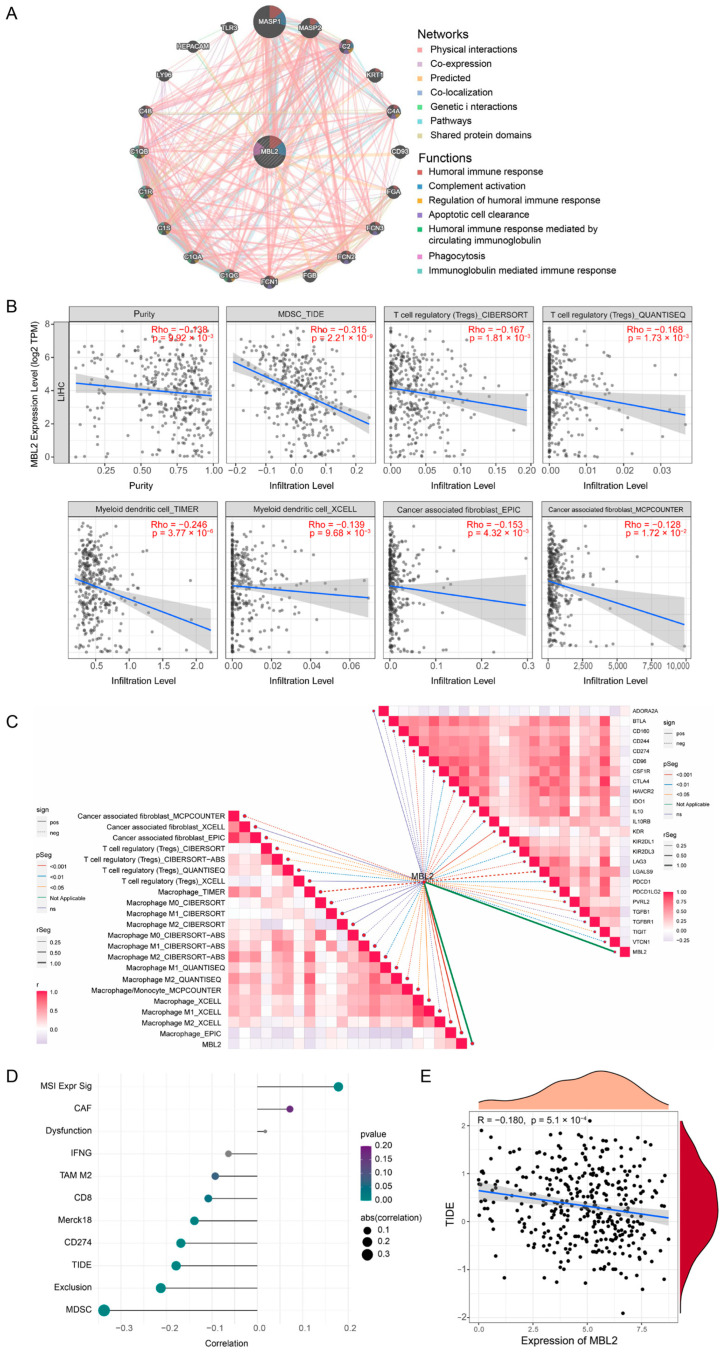
MBL2 expression is associated with immune infiltration of HCC. (**A**) Protein–protein interaction network and functional annotation for MBL2. (**B**) TIMER 2.0 was employed to analyze the correlations between MBL2 expression and immune infiltration of cancer-associated fibroblasts, T-regulatory cells, natural killer cells, and tumor purity. (**C**) The ImmuCellAI platform was used to assess the correlations among MBL2 expression, immune-suppressive genes, and infiltration of diverse immune cells. (**D**,**E**) Correlation analyses were performed using the TIDE method to examine the relationships between MBL2 expression and various immune features. MBL2, mannose-binding lectin 2; HCC, hepatocellular carcinoma; TIDE, tumor immune dysfunction and exclusion.

Furthermore, we used the GeneMANIA online portal to create a protein–protein interaction (PPI) network for MBL2. As shown in Figure 6A, MBL2 showed significant physical interactions with MASP1, MASP2, KRT1, FCN3, etc., which were similar to the PPI results from STRING (Figure S3C). The functional enrichment of these network proteins indicated that MBL2 was primarily related to physiological immune processes, including the humoral immune response, complement activation, and apoptotic cell clearance (Figure 6A), which indicated that MBL2, a secretory protein, could potentially interact with the immune microenvironment. To further validate the above conjecture, we first used TIMER 2.0 to evaluate the effect of MBL2 on the tumor immune microenvironment of HCC. As displayed in Figure 6B, MBL2 expression had a negative influence on tumor purity and the immunosuppressive microenvironment was infiltrated by MDSC, CAF, dendritic cells, and Tregs. To further validate this result, we utilized the ImmuCellAI platform to calculate immune cell infiltration in HCC from the TCGA cohort. A correlation analysis was conducted to assess the impact of MBL2. The findings demonstrated a significant negative correlation between MBL2 expression and the infiltration of CD8-naïve T cells, B cells, and natural regulatory T cells (Tregs), while a significant positive correlation was observed between MBL2 expression and the infiltration of Th17 and CD8 T cells (Figure 6C). A gene coexpression analysis was conducted to investigate the correlation between MBL2 expression and immunosuppressive genes in HCC. Most immunosuppressive genes exhibited a clear negative association with MBL2 expression, except for ADORA2A (Figure 6C). These findings suggest that the reduced secretion of MBL2 is linked to an immunosuppressive environment in HCC. TIDE, a bioinformatics algorithm that models two key mechanisms of tumor immune evasion, is widely used to assess immune infiltration associated with the response to immune therapy. Consequently, we conducted additional analyses to explore the relationships among MBL2 expression, the immune therapy response score, and the immune infiltration quantified by TIDE. MBL2 expression was significantly negatively correlated with MDSC infiltration, immune exclusion subtypes, and the TIDE score (Figure 6D,E).

## 4. Discussion

In the current study, we conducted high-throughput sequencing on HCC patients with both high and low CTC expression, followed by a comprehensive multi-omics analysis to elucidate the significance of MBL2. We assessed the clinical prognostic significance of MBL2 and delved into potential upstream miRNA regulatory mechanisms as well as downstream pathways linked to MBL2. Furthermore, we scrutinized the influence of MBL2 on immune infiltration within the HCC microenvironment. Additionally, we conducted validation experiments to confirm the inhibitory impact of MBL2 on HCC progression in vitro and in vivo. Recognizing the pivotal role of miRNAs in cancer regulation, we substantiated the downregulation of miR-34c-3p targeting MBL2. We systematically elucidated the significance of MBL2, thereby establishing the foundation for future research exploring MBL2 as a potential therapeutic target for HCC.

The C-terminal carbohydrate-recognition domain of the MBL2 protein can widely recognize pathogen-associated molecular patterns of various pathogens and apoptotic cells, activate the complement system, promote phagocytosis and immune regulation, and inhibit and neutralize viruses [34]. Nonetheless, MBL is a critical mucosal surface defense molecule. The collagen-like region of MBL binds to mannan-associated serine protease, activates the complement lectin pathway to exert an indirect immunomodulatory effect, or binds to the phagocyte agglutinin receptor to exert a direct opsonizing effect [17]. This functional structure is crucial in anti-infection processes and apoptotic cell clearance.

Genetic variants of MBL2 are associated with the progression of ovarian, gastric, lung, and breast cancer [18,35,36,37,38]. Baccarelli et al. studied the relationships between MBL variants and the risk of gastric cancer and found that the HYD haplotype had increased risk compared to the HYA haplotype [38]. Chronic HBV or hepatitis C virus (HCV) infection has been associated with low MBL levels in previous liver-cancer-related studies. Furthermore, several studies have demonstrated that the MBL2 polymorphism is associated with HBV and HCV infections as well as the risk of HCC [16,39]. Previous studies have indicated the significant involvement of MBL2 in tumor development, including HCC. However, the underlying mechanism of the impact of MBL2 on HCC progression remains poorly understood.

MBL2 is closely related to CTC formation and is a potent marker of tumor micrometastases and progression. Moreover, MBL2 is underexpressed in HCC, and a low expression level of MBL2 is a risk factor for poor prognosis in HCC patients. To test this hypothesis, we simultaneously analyzed datasets from public platforms and found that MBL2 was downregulated in liver cancer specifically. Additionally, notable disparities were observed in the expression of MBL2 among various molecular subtypes. Subsequently, we performed a GSEA to explore the potential mechanism by which MBL2 affects HCC progression. Our findings revealed a correlation between low MBL2 expression and the activation of various malignant pathways, including the cell cycle [40], DNA replication, miRNAs in cancer, the vascular endothelial growth factor signaling pathway [41], and the cancer stem cell-related pathway [42]. In addition, we combined in vivo and cellular experiments to confirm that low MBL2 expression promotes the basic oncogenic functions of hepatoma cells.

We investigated potential upstream miRNAs regulating MBL2 to unravel the mechanism underlying its dysregulation. Using the DIANA and TargetScan databases, we predicted miRNAs targeting MBL2. Using TCGA’s liver cancer tissue miRNA database, we discovered a significant correlation between MBL2 and miR-34c-3p. High expression levels of miR-34c-3p in tumor tissue and poor prognosis suggest its promotion of HCC development and progression. We verified the promoting effect of miR-34c-3p in hepatoma cells and predicted its target on MBL2 through bioinformatics, suggesting miR-34c-3p targeting MBL2 promotes HCC development.

MBL2 mainly acts as a secreted protein and exerts its biological functions in an exocrine manner [43]. MBL2 is implicated in the host immune response, indicating potential crosstalk between MBL2 and the immune cell infiltration of the tumor microenvironment. Therefore, we analyzed the association between MBL2 and lymphocytes in the tumor microenvironment. Our findings revealed a significant correlation between low MBL2 expression and the expression of numerous immunosuppressive genes. Moreover, low MBL2 expression was associated with enhanced infiltration of immunosuppressive cells, including Tregs [44], M2 macrophages [45], and MDSCs [46]. High MBL2 expression was correlated with antitumor immune cells, including natural killer cells [47] and CD8 T cells [48], indicating a close relationship between low MBL2 expression and tumor immune escape. Interestingly, MBL can be generated using either plasma-derived or recombinant methods. Several clear clinical scenarios exist for MBL2 replacement trials, including MBL deficiency in cases of recurrent childhood infection, prophylaxis against sepsis following chemotherapy, and rapidly progressive cystic fibrosis. Our results indicate that MBL replacement has potential therapeutic value for enhancing immunotherapy in HCC. Admittedly, we placed an emphasis on descriptive observations in the absence of novel and rigorous mechanistic studies. Further research should be undertaken to explore downstream mechanisms through which MBL2 might regulate the development of HCC.

## 5. Conclusions

In conclusion, our findings revealed the regulatory role of MBL2 in HCC proliferation, migration, and invasion. Based on MBL2-related miRNAs, we validated the interaction between MBL2 and miR-34c-3p. Our results further elucidate why decreased MBL2 expression in hepatocytes is a key indicator of poor prognosis. Meanwhile, miR-34c-3p was found to be an upstream mechanism of the downregulation of MBL2 expression and could be a therapeutic target, expanding treatment options for patients with HCC.

## Data Availability

All datasets were sourced from publicly accessible databases and published studies. Additional data generated during this study, as well as the code used, can be obtained from the corresponding authors.

## Supplementary Materials

The following supporting information can be downloaded at https://www.mdpi.com/article/10.3390/cancers15194900/s1, Figure S1: Multi-omics analysis of MBL2 and Multivariate Cox regression analyse of MBL2 with other clinical parameters; Figure S2: Heat map and pathway activation chart; Figure S3: The calculated intensity ratios of Western blot bands and protein–protein interaction network analysis for MBL2 in STRING online portal; Table S1: Sequences of the miRNA mimics and negative control; Table S2: Sequences of the miRNA inhibitor and negative control; Table S3: Sequences of the constructed plasmids and the mutation sites; Table S4: Coexpression correlation analysis of MBL2 using the LinkedOmics dataset.
