# Supplementary material for: Mannose-Binding Lectin 2 as a Potential Therapeutic Target for Hepatocellular Carcinoma: Multi-Omics Analysis and Experimental Validation
Source: Cancers (Basel). 2023 Oct 9;15(19):4900. doi: 10.3390/cancers15194900 (PMC10571644; doi:10.3390/cancers15194900)
Supplement: Supplementary file 1 [file cancers-15-04900-s001.zip › Supplementary_Figure_S1.pdf]

Supplementary Material

Mannose-Binding Lectin 2 as a Potential Therapeutic Target for Hepatocellular Carcinoma: Multi-Omics Analysis and Experimental Validation

Hangyu Liao, Jun Yang, Yuyan Xu, Juncheng Xie, Ke Li, Kunling Chen, Jingyuan Pei, Qiong Luo, Mingxin Pan*

*** Correspondence: Mingxin Pan; panmx@smu.edu.cn**

Supplementary Figure S1. (A) ANTE database indicated that the expression of MBL2 in the liver is relatively higher compared to other normal human tissues. (B) The mRNA expression of MBL2 in tumor and paired adjacent liver tissues from the HBV-HCC cohort was analyzed. (C) MBL2 expression was significantly lower in the proliferation than in the metabolism and microenvironment dysregulated subgroups. (D-E) Survival analysis of HCC patients based on the expression of MBL2, 324 including the ICGC database (D) and the HBV-HCC cohort (E). (F) Multivariate Cox regression analyse of MBL2 with other clinical parameters in the HBV-HCC cohort.

ANTE, atlas of normal tissue expression; MBL2, mannose-binding lectin 2; TCGA, The Cancer Genome Atlas; HBV-HCC, hepatitis B virus-related hepatocellular carcinoma

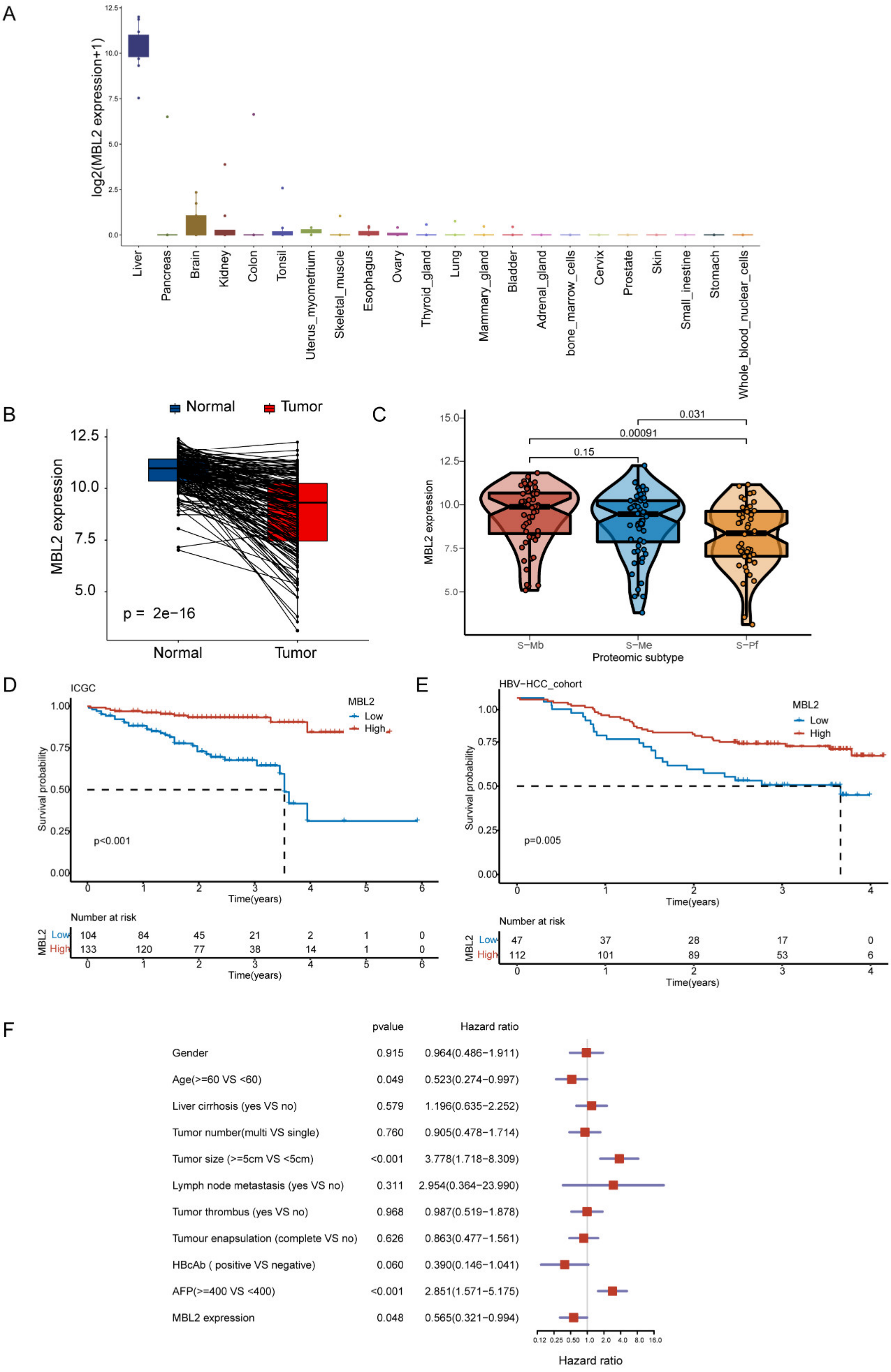
A
log2(MBL2 expression+1)
Liver
Pancreas
Brain
Kidney
Colon
Tonsil
Uterus_myometrium
Skeletal_muscle
Esophagus
Ovary
Thyroid_gland
Lung
Mammary_gland
Bladder
Adrenal_gland
bone_marrow_cells
Cervix
Prostate
Skin
Small_inestine
Stomach
Whole_blood_nuclear_cells
B
Normal
Tumor
MBL2 expression
p = 2e−16
C
0.031
0.00091
0.15
MBL2 expression
S−Mb
S−Me
S−Pf
Proteomic subtype
D
ICGC
MBL2
Low
High
Survival probability
p<0.001
Time(years)
Number at risk
Low 104 84 45 21 2 1 0
High 133 120 77 38 14 1 0
E
HBV−HCC_cohort
MBL2
Low
High
Survival probability
p=0.005
Time(years)
Number at risk
Low 47 37 28 17 0
High 112 101 89 53 6
F
pvalue
Hazard ratio
Gender 0.915 0.964(0.486−1.911)
Age(>=60 VS <60) 0.049 0.523(0.274−0.997)
Liver cirrhosis (yes VS no) 0.579 1.196(0.635−2.252)
Tumor number(multi VS single) 0.760 0.905(0.478−1.714)
Tumor size (>=5cm VS <5cm) <0.001 3.778(1.718−8.309)
Lymph node metastasis (yes VS no) 0.311 2.954(0.364−23.990)
Tumor thrombus (yes VS no) 0.968 0.987(0.519−1.878)
Tumour enapsulation (complete VS no) 0.626 0.863(0.477−1.561)
HBcAb (positive VS negative) 0.060 0.390(0.146−1.041)
AFP(>=400 VS <400) <0.001 2.851(1.571−5.175)
MBL2 expression 0.048 0.565(0.321−0.994)
Hazard ratio
