# Supplementary material for: Mannose-Binding Lectin 2 as a Potential Therapeutic Target for Hepatocellular Carcinoma: Multi-Omics Analysis and Experimental Validation
Source: Cancers (Basel). 2023 Oct 9;15(19):4900. doi: 10.3390/cancers15194900 (PMC10571644; doi:10.3390/cancers15194900)
Supplement: Supplementary file 1 [file cancers-15-04900-s001.zip › Supplementary_Figure_S2.pdf]

Supplementary Material

Mannose-Binding Lectin 2 as a Potential Therapeutic Target for Hepatocellular Carcinoma: Multi-Omics Analysis and Experimental Validation

Hangyu Liao, Jun Yang, Yuyan Xu, Juncheng Xie, Ke Li, Kunling Chen, Jingyuan Pei, Qiong Luo, Mingxin Pan*

*** Correspondence: Mingxin Pan: panmx@smu.edu.cn**

Supplementary Figure S2. (A) Heat map illustrating the top 50 genes that exhibit negative correlation with MBL2. (B-C) Significantly enriched KEGG pathways and transcription factor targets of the genes coexpressed with MBL2 in HCC. (D) Pathway activation chart. Green lines show top 10 most strongly activated pathways (ordered top to bottom), red lines show top 10 most strongly inhibited pathways (ordered bottom to top). Thickness is proportionate to absolute value of PAL. (E) Analysis of differential expression of apoptosis-related biomarkers between distinct MBL2 expression cohorts in the TCGA-LIHC dataset.

MBL2, mannose-binding lectin 2; KEGG, Kyoto Encyclopedia of Genes and Genomes; TCGA-LIHC, The Cancer Genome Atlas-Liver Hepatocellular Carcinoma

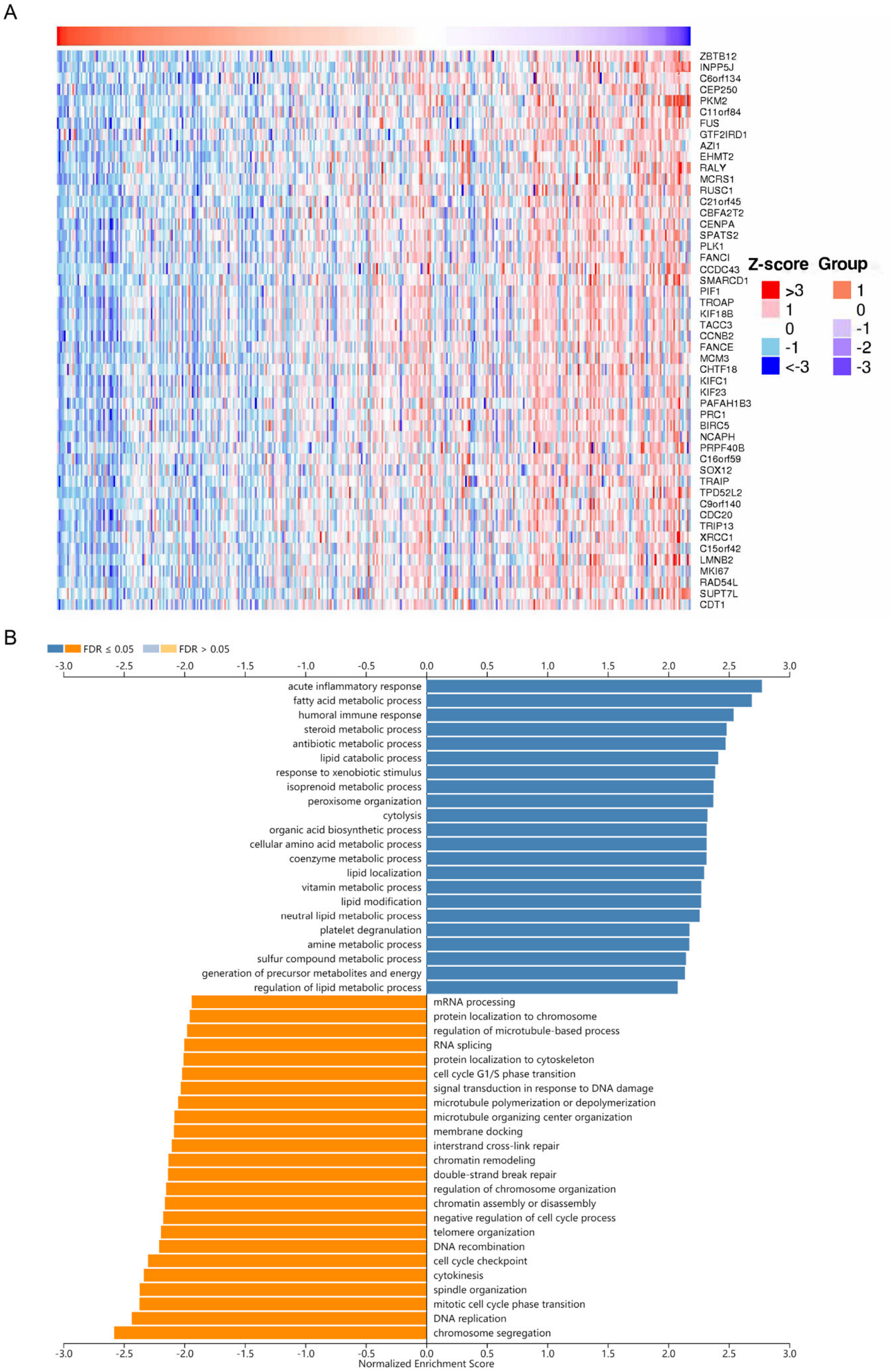
A
ZBTB12
INPP5J
C6orf134
CEP250
PKM2
C11orf84
FUS
GTF2IRD1
AZI1
EHMT2
RALY
MCRS1
RUSC1
C21orf45
CBFA2T2
CENPA
SPATS2
PLK1
FANCI
CCDC43
SMARCD1
PIF1
TROAP
KIF18B
TACC3
CCNB2
FANCE
MCM3
CHTF18
KIFC1
KIF23
PAFAH1B3
PRC1
BIRC5
NCAPH
PRPF40B
C16orf59
SOX12
TRAIP
TPD52L2
C9orf140
CDC20
TRIP13
XRCC1
C15orf42
LMNB2
MKI67
RAD54L
SUPT7L
CDT1
Z-score
>3
1
0
-1
<-3
Group
1
0
-1
-2
-3
B
FDR ≤ 0.05
FDR > 0.05
-3.0
-2.5
-2.0
-1.5
-1.0
-0.5
0.0
0.5
1.0
1.5
2.0
2.5
3.0
acute inflammatory response
fatty acid metabolic process
humoral immune response
steroid metabolic process
antibiotic metabolic process
lipid catabolic process
response to xenobiotic stimulus
isoprenoid metabolic process
peroxisome organization
cytolysis
organic acid biosynthetic process
cellular amino acid metabolic process
coenzyme metabolic process
lipid localization
vitamin metabolic process
lipid modification
neutral lipid metabolic process
platelet degranulation
amine metabolic process
sulfur compound metabolic process
generation of precursor metabolites and energy
regulation of lipid metabolic process
mRNA processing
protein localization to chromosome
regulation of microtubule-based process
RNA splicing
protein localization to cytoskeleton
cell cycle G1/S phase transition
signal transduction in response to DNA damage
microtubule polymerization or depolymerization
microtubule organizing center organization
membrane docking
interstrand cross-link repair
chromatin remodeling
double-strand break repair
regulation of chromosome organization
chromatin assembly or disassembly
negative regulation of cell cycle process
telomere organization
DNA recombination
cell cycle checkpoint
cytokinesis
spindle organization
mitotic cell cycle phase transition
DNA replication
chromosome segregation
Normalized Enrichment Score

C

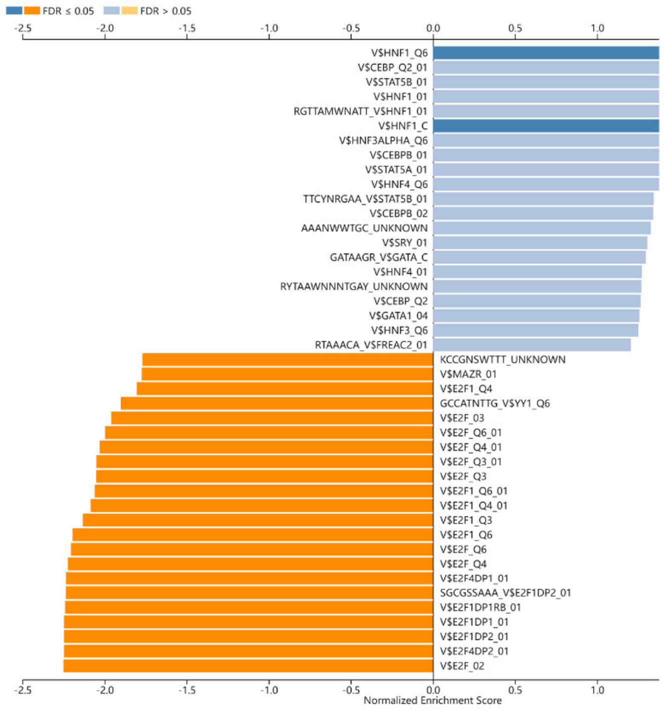
FDR ≤ 0.05
FDR > 0.05
-2.5 -2.0 -1.5 -1.0 -0.5 0.0 0.5 1.0
V$HNF1_Q6
V$CEBP_Q2_01
V$STAT5B_01
V$HNF1_01
RGTTAMWNATT_V$HNF1_01
V$HNF1_C
V$HNF3ALPHA_Q6
V$CEBPB_01
V$STAT5A_01
V$HNF4_Q6
TTCYNRGAA_V$STAT5B_01
V$CEBPB_02
AAANWWTGC_UNKNOWN
V$SRY_01
GATAAGR_V$GATA_C
V$HNF4_01
RYTAAWNNNTGAY_UNKNOWN
V$CEBP_Q2
V$GATA1_04
V$HNF3_Q6
RTAAACA_V$FREAC2_01
KCCGNSWTTT_UNKNOWN
V$MAZR_01
V$E2F1_Q4
GCCATNTTG_V$YY1_Q6
V$E2F_03
V$E2F_Q6_01
V$E2F_Q4_01
V$E2F_Q3_01
V$E2F_Q3
V$E2F1_Q6_01
V$E2F1_Q4_01
V$E2F1_Q3
V$E2F1_Q6
V$E2F_Q6
V$E2F_Q4
V$E2F4DP1_01
SGCGSSAAA_V$E2F1DP2_01
V$E2F1DP1RB_01
V$E2F1DP1_01
V$E2F1DP2_01
V$E2F4DP2_01
V$E2F_02
Normalized Enrichment Score

D

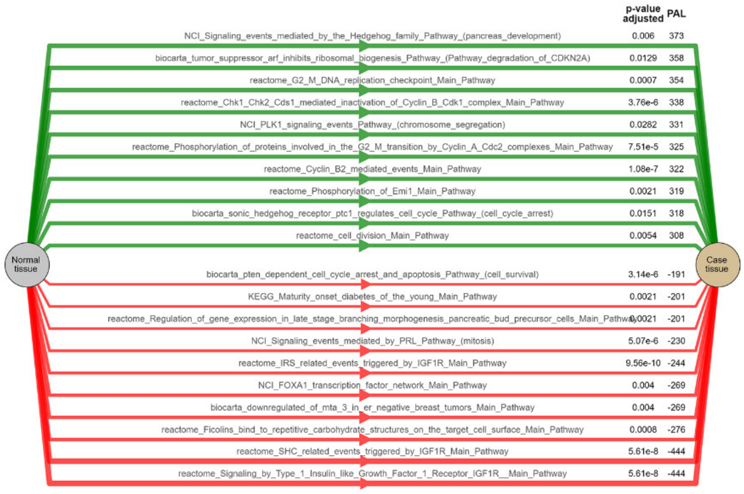
p-value adjusted
PAL
Normal tissue
Case tissue
NCI_Signaling_events_mediated_by_the_Hedgehog_family_Pathway_(pancreas_development) 0.006 373
biocarta_tumor_suppressor_arf_inhibits_ribosomal_biogenesis_Pathway_(Pathway_degradation_of_CDKN2A) 0.0129 358
reactome_G2_M_DNA_replication_checkpoint_Main_Pathway 0.0007 354
reactome_Chk1_Chk2_Cds1_mediated_inactivation_of_Cyclin_B_Cdk1_complex_Main_Pathway 3.76e-6 338
NCI_PLK1_signaling_events_Pathway_(chromosome_segregation) 0.0282 331
reactome_Phosphorylation_of_proteins_involved_in_the_G2_M_transition_by_Cyclin_A_Cdc2_complexes_Main_Pathway 7.51e-5 325
reactome_Cyclin_B2_mediated_events_Main_Pathway 1.08e-7 322
reactome_Phosphorylation_of_Emi1_Main_Pathway 0.0021 319
biocarta_sonic_hedgehog_receptor_ptc1_regulates_cell_cycle_Pathway_(cell_cycle_arrest) 0.0151 318
reactome_cell_division_Main_Pathway 0.0054 308
biocarta_pten_dependent_cell_cycle_arrest_and_apoptosis_Pathway_(cell_survival) 3.14e-6 -191
KEGG_Maturity_onset_diabetes_of_the_young_Main_Pathway 0.0021 -201
reactome_Regulation_of_gene_expression_in_late_stage_branching_morphogenesis_pancreatic_bud_precursor_cells_Main_Pathway 0.0021 -201
NCI_Signaling_events_mediated_by_PRL_Pathway_(mitosis) 5.07e-6 -230
reactome_IRS_related_events_triggered_by_IGF1R_Main_Pathway 9.56e-10 -244
NCI_FOXA1_transcription_factor_network_Main_Pathway 0.004 -269
biocarta_downregulated_of_mta_3_in_er_negative_breast_tumors_Main_Pathway 0.004 -269
reactome_Ficolins_bind_to_repetitive_carbohydrate_structures_on_the_target_cell_surface_Main_Pathway 0.0008 -276
reactome_SHC_related_events_triggered_by_IGF1R_Main_Pathway 5.61e-8 -444
reactome_Signaling_by_Type_1_Insulin_like_Growth_Factor_1_Receptor_IGF1R__Main_Pathway 5.61e-8 -444

E

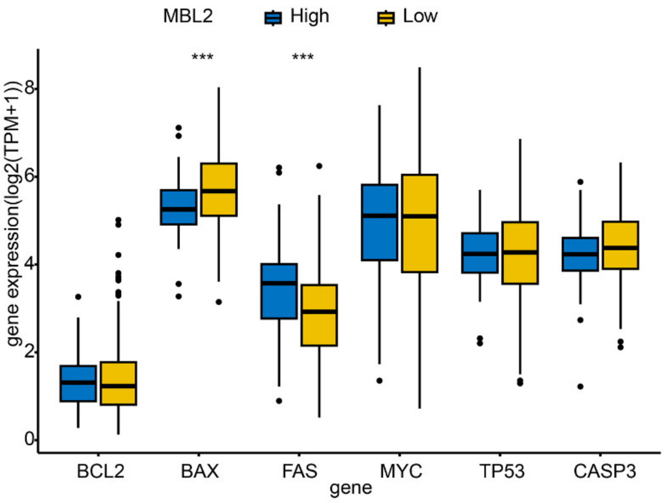
MBL2
High
Low
***
***
gene expression(log2(TPM+1))
0
2
4
6
8
BCL2
BAX
FAS
MYC
TP53
CASP3
gene
