# Supplementary material for: Mannose-Binding Lectin 2 as a Potential Therapeutic Target for Hepatocellular Carcinoma: Multi-Omics Analysis and Experimental Validation
Source: Cancers (Basel). 2023 Oct 9;15(19):4900. doi: 10.3390/cancers15194900 (PMC10571644; doi:10.3390/cancers15194900)

Supplementary Material

Mannose-Binding Lectin 2 as a Potential Therapeutic Target for Hepatocellular Carcinoma: Multi-Omics Analysis and Experimental Validation

Hangyu Liao, Jun Yang, Yuyan Xu, Juncheng Xie, Ke Li, Kunling Chen, Jingyuan Pei, Qiong Luo, Mingxin Pan*

*** Correspondence: Mingxin Pan: panmx@smu.edu.cn**

Supplementary Figure S3. (A) The calculated intensity ratios of Western blot bands indicated that the protein expression of MBL2 was increased in the LV-MBL2 group compared to the control group in both Huh7 and BEL-7404 cell lines. (B) In both Huh7 and BEL-7404 cell lines, the translational expression of MBL2 was reduced in the miR-34c-3p mimics group compared to the control group. (C) Protein–protein interaction network analysis for MBL2 in STRING online portal.

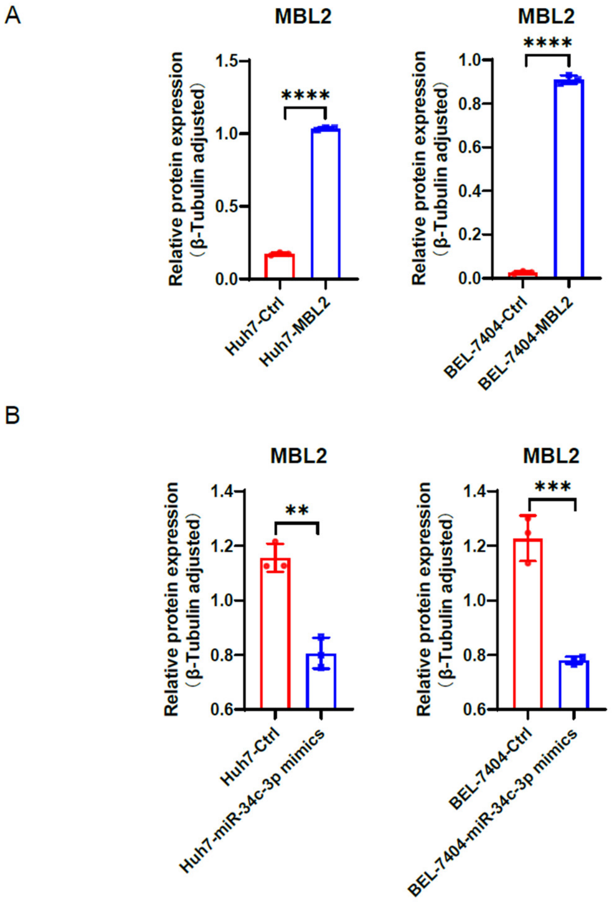

C

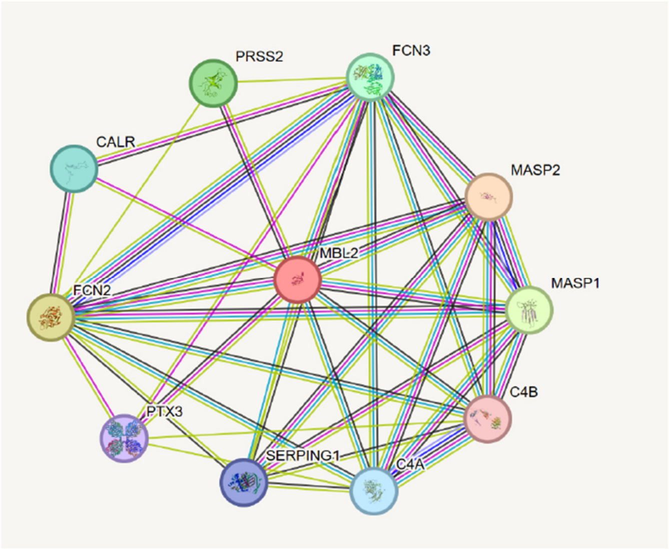

Supplement: Supplementary file 1 [file cancers-15-04900-s001.zip › Supplementary_Figure_S3.pdf]
