# Supplementary material for: Mannose-Binding Lectin 2 as a Potential Therapeutic Target for Hepatocellular Carcinoma: Multi-Omics Analysis and Experimental Validation
Source: Cancers (Basel). 2023 Oct 9;15(19):4900. doi: 10.3390/cancers15194900 (PMC10571644; doi:10.3390/cancers15194900)
Supplement: Supplementary file 1 [file cancers-15-04900-s001.zip › Supplementary_Tables_S1-S3.pdf]

# *Supplementary Material*

## Mannose-Binding Lectin 2 as a Potential Therapeutic Target for Hepatocellular Carcinoma: Multi-Omics Analysis and Experimental Validation

**Hangyu Liao, Jun Yang, Yuyan Xu, Juncheng Xie, Ke Li, Kunling Chen, Jingyuan Pei, Qiong Luo, Mingxin Pan***

*** Correspondence: Mingxin Pan: panmx@smu.edu.cn**

**1. Supplementary Tables**

**Supplementary Table S1:** Sequences of the miRNA mimics and negative control

| RNA oligo | Sequences |
|---|---|
| miR-34c-3p mimics | AAU CAC UAA CCA CAC GGC CAGG |
| | UGG CCG UGU GGU UAG UGA UUUU |
| Negative control | UUC UCC GAA CGU GUC ACG UTT |
| | ACG UGA CAC GUU CGG AGA ATT |

**Supplementary Table S2:** Sequences of the miRNA inhibitor and negative control

| RNA oligo | Sequences |
|---|---|
| miR-34c-3p inhibitor | CCUGGCCGUGUGGUUAGUGAUU |
| MiRNA inhibitor negative control | CAGUACUUUUGUGUAGUACAA |

**Supplementary Table S3:** Sequences of the constructed plasmids and the mutation sites

| Name | sequences |
|---|---|
| MBL2-WT | GCTAGCTTTCTACAAAATCCTGATGAGGTTTCTACTGGGACCACATTGAGTCTATCAATCAACTT<br>ATGCAGAACTGGCAACTTACTACTGAATCTCTAATCAATGTTCATCATGTATCGCTTCATGTAAC<br>TAGAATTTCTTTAACTTAATTGCTATGTTTTGACATTTTTAGTTTAAAAACCTTGTATATCTTGT<br>TTTGGTGGTTTTAGTGATTTTAATAATATATTTTAAATATTTTTTCTTTTCTATTGTTGTACACA<br>GAAATACAGTTAAGTTTTGTGTGTAGTCTTACGATGTTTAGTAAACTCAATAAGTTTATTTCTTA<br>AATCTAGTAATTTGTAGATTCCTCTGGATTTTGTATATGCATAGTCATGTAAGCTGAAAATATGG<br>CAATACTTGCTTCTTCCCAAGTCGAC |
| MBL2-Mut | GCTAGCTTTCTACAAAATCCTGATGAGGTTTCTACTGGGACCACATTGAGTCTATCAATCAACTT<br>ATGCAGAACTGGCAACTTACTACTGAATCTCTAATCAATGTTCATCATGTATCGCTTCATGTAAC<br>TAGAATTTCTTTAACTTAATTGCTATGTTTTGACATTTTTAGTTTAAAAACCTTGTATATCTTGT<br>TTTGGTGGTTTGATTAATTTTAATAATATATTTTAAATATTTTTTCTTTTCTATTGTTGTACACA<br>GAAATACAGTTAAGTTTTGTGTGTAGTCTTACGATGTTTAGTAAACTCAATAAGTTTATTTCTTA<br>AATCTAGTAATTTGTAGATTCCTCTGGATTTTGTATATGCATAGTCATGTAAGCTGAAAATATGG<br>CAATACTTGCTTCTTCCCAAGTCGAC |
