# Supplementary figures and images for: Mannose-Binding Lectin 2 as a Potential Therapeutic Target for Hepatocellular Carcinoma: Multi-Omics Analysis and Experimental Validation
Source: Cancers (Basel). 2023 Oct 9;15(19):4900. doi: 10.3390/cancers15194900 (PMC10571644; doi:10.3390/cancers15194900)

Figure 3-C

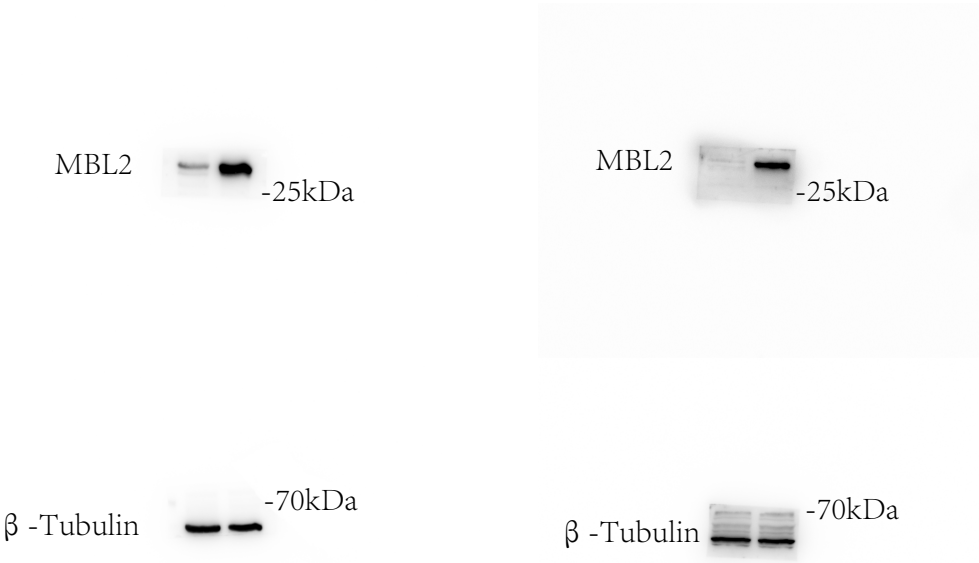

Figure 4-I

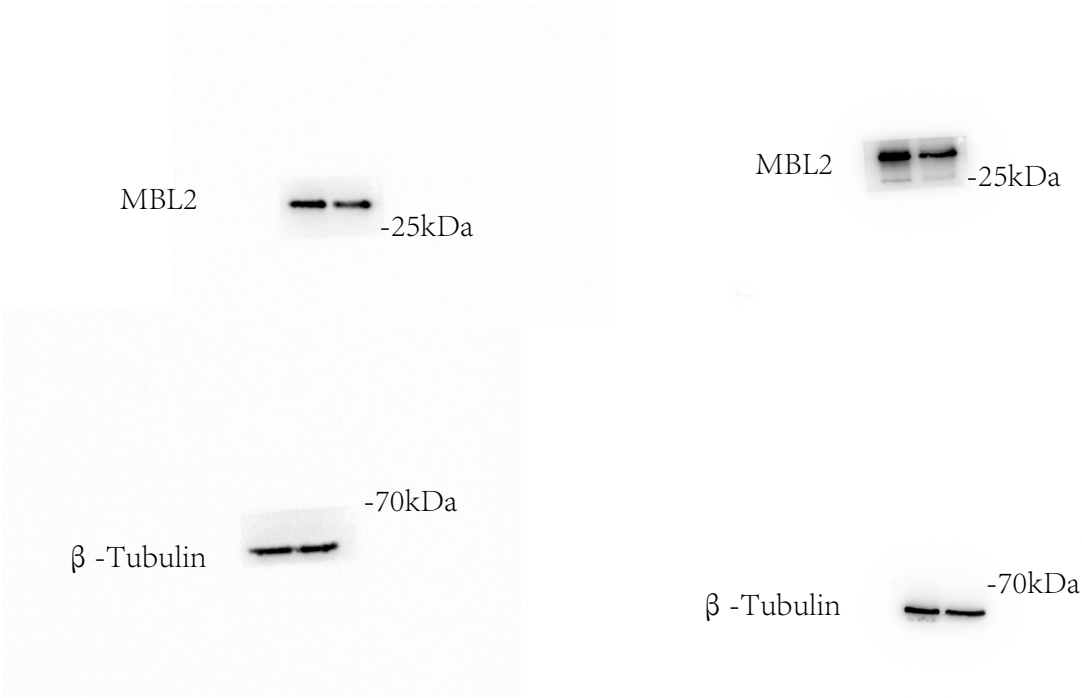

Supplement: Supplementary file 1 [file cancers-15-04900-s001.zip › WB-raw data.pdf]
